# *B2M* drives PD-1 inhibitor resistance in DLBCL through independent dual immune escape mechanisms

**DOI:** 10.1515/biol-2025-1226

**Published:** 2026-04-20

**Authors:** Ying Liu, Xiao Xu, Chengtian Li, Shangdong Mou, Feifei Wang, Zhongqiang Yao, Qingjuan Chen

**Affiliations:** Department of Oncology, 3201 Hospital, Hanzhong 723000, China; Department of Hematology, 3201 Hospital, Hanzhong 723000, China

**Keywords:** DLBCL, PD-1 inhibitor resistance, β2-microglobulin, MHC-I, PD-L1

## Abstract

PD-1/PD-L1 immune checkpoint inhibitors (ICIs) demonstrate promising therapeutic potential in diffuse large B-cell lymphoma (DLBCL). However, a subset of DLBCL patients exhibits primary or acquired resistance to PD-1 blockade. This study investigated the mechanistic role of β2-microglobulin (*B2M*) mutations in modulating PD-L1 expression and their contribution to ICI resistance in DLBCL. The association between *B2M* mutations and PD-L1 expression in DLBCL patient samples was analyzed via immunohistochemistry. A *B2M*-knockout DLBCL cell line model was established, followed by evaluation of PD-L1 and MHC-I expression changes through qRT-PCR, western blot, and flow cytometry. *T* cell activation (CD69/CD25 markers) and cytotoxic activity (lactate dehydrogenase release) were assessed in PBMC co-culture experiments. Mechanistic studies employing MHC-I inhibitors and IFN-*γ* stimulation were conducted to dissect the regulatory relationship between MHC-I and PD-L1. Co-culture systems with PD-1 blockade was performed to validate the impact of *B2M* on PD-1 inhibitor resistance. The results demonstrated that *B2M* knockout significantly downregulated both PD-L1 mRNA and protein levels, concomitant with impaired MHC-I complex assembly and reduced TAP1/2 expression. *B2M*-deficient cells failed to activate CD8+ *T* cells, evidenced by diminished CD69^+^/CD25^+^ surface expression and reduced cytotoxic efficiency. Notably, *B2M* ablation abolished IFN-γ-induced PD-L1 upregulation (*p* < 0.05), demonstrating that intact MHC-I functionality is a prerequisite for PD-L1 expression. Further co-culture systems with PD-1 blockade validated the functional roles of MHC-I and PD-L1 in modulating PD-1 inhibitor resistance mediated by *B2M*. In conclusion, this work indicated that *B2M* mutations synergistically drive DLBCL resistance to PD-1 inhibitors through dual mechanisms-suppressing PD-L1 expression and completely disabling antigen presentation. The study highlights that combined strategies restoring MHC-I expression and targeting PD-L1 may overcome immunotherapy resistance, offering a novel direction for precision therapy in DLBCL.

## 1 Introduction

Diffuse large B-cell lymphoma (DLBCL) constitutes the most prevalent subtype of non-Hodgkin lymphoma (NHL), accounting for approximately 35 % of all NHL cases worldwide. In 2016 alone, the United States reported an estimated 27,650 new DLBCL diagnoses, underscoring its significant clinical burden [1]. The current standard frontline therapy for DLBCL patients involves the R–CHOP regimen, which combines rituximab with cyclophosphamide, doxorubicin, vincristine, and prednisone. While this treatment modality has significantly improved patient outcomes, disease relapse still occurs in 30 %–40 % of cases [2], 3]. Immune checkpoint inhibitors (ICIs) (e.g. PD-1/PD-L1 inhibitor) are primarily used as salvage therapy for relapsed/refractory DLBCL, particularly in patients resistant to R–CHOP regimens or unable to tolerate intensive chemotherapy, achieving an objective response rate of 30 %–40 % [4], 5]. However, a subset of patients exhibits intrinsic or acquired resistance to PD-1/PD-L1 blockade, with mechanisms not yet fully elucidated. This knowledge gap not only hinders patient stratification but also limits the long-term therapeutic benefits.

The therapeutic success of immune checkpoint blockade hinges on the antitumor activity of CD8^+^ *T* cells. However, this CD8^+^ *T* cell-dependent efficacy requires functional antigen presentation by tumor cells – a vulnerability that drives the evolution of resistance mechanisms [6], 7]. β2-microglobulin (*B2M*), the *β*-chain of MHC class I (MHC-I) molecules, serves as an essential component for MHC-I-mediated antigen processing and presentation [8]. Neoantigen-derived peptides bind to MHC-I complexes formed by the MHC-I *α* chain (HLA-I) and *B2M*, with the resulting complexes displayed on the cell surface and recognized by CD8^+^ *T* cells via *T*-cell receptors (TCRs) [8]. In the absence of *B2M*, peptide-MHC-I complexes fail to assemble and present antigenic signals, resulting in impaired CD8^+^ *T* cell activation and resistance to PD-1/PD-L1 immunotherapy [7]. The critical role of *B2M* gene deficiency in immunotherapy resistance was first identified through observations in melanoma patients who developed resistance to PD-1 inhibitors [9]. *B2M* mutations were also identified in two lung cancer patients exhibiting resistance to PD-1 inhibitors, and these genetic alterations are postulated to be a primary driver of anti-PD-1 therapy resistance [10]. Nevertheless, the role of *B2M* in driving PD-1 blockade resistance among DLBCL patients remains inadequately investigated.

In this study, we observed reduced PD-L1 expression in tumor tissues of DLBCL patients harboring *B2M* mutations. Further mechanistic investigations revealed that *B2M* defects may drive resistance to PD-1 immunotherapy by suppressing both PD-L1 expression and MHC-I-mediated antigen presentation. Collectively, our findings identify *B2M* as a novel therapeutic target and provide a rationale for combinatorial strategies to enhance immunotherapy efficacy in DLBCL.

## 2 Materials and methods

### 2.1 Experiment protocol

We first performed immunohistochemical (IHC) staining to detect PD-L1 expression in DLBCL patients with *B2M* gene mutations. Subsequently, we established *B2M*-knockout DLBCL cell line models to investigate the correlation between *B2M* deficiency and PD-L1/MHC-I expression. Followed by co-culturing *B2M*-deficient DLBCL cells with peripheral blood mononuclear cells (PBMC), we evaluated the impact of *B2M* mutation on T-cell activation (using CD69/CD25 marker) and cytotoxic activity (assessed through lactate dehydrogenase (LDH) release assay). Moreover, employing MHC-I inhibitor and intervention with IFN-γ in DLBCL cells, we examined the regulatory relationship between MHC-I and PD-L1 expression. Finally, we co-cultured wild-type and *B2M*-knockdown DLBCL cells with PBMCs, adding clinically relevant doses of anti-PD-1 antibodies along with MHC-I or PD-L1 overexpression vectors to validate the impact of *B2M* on PD-1 resistance and its underlying mechanisms.

### 2.2 Patients

Patients with histopathologically confirmed *de novo* DLBCL, classified according to the World Health Organization criteria, were prospectively enrolled. The cohort was stratified into two groups (*n* = 6 per group): (1) an experimental cohort harboring *B2M* mutations, and (2) a control cohort consisting of age-matched counterparts without *B2M* alterations. Exclusion criteria encompassed individuals with concurrent hematological malignancies, solid tumors, or autoimmune disorders.

**Informed consent:** Informed consent has been obtained from all individuals included in this study.

**Ethical approval:** The research related to human use has been complied with all the relevant national regulations, institutional policies and in accordance with the tenets of the Helsinki Declaration, and has been approved by the Institutional Review Board of 3,201 Hospital (approval number: 2024-016).

### 2.3 Immunohistochemical staining

The obtained tissue specimens were dehydrated through a graded ethanol series, followed by clearing, paraffin infiltration, and embedding. The sections were cut using a Leica rotary microtome and baked at 60 °C for 3. After dewaxing, the sections underwent antigen retrieval and endogenous peroxidase blocking before being incubated with blocking serum for 30 min. Primary antibody incubation (rabbit monoclonal anti-PD-L1, Abcam, ab205921) was performed overnight at 4 °C, followed by secondary antibody (Agilent, K5007) incubation at 37 °C for 30 min. Subsequent steps included DAB chromogenic development, hematoxylin (Keycell, BT-P107) counterstaining, dehydration, and mounting. Finally, stained sections were visualized and imaged under a microscope (Olympus, BX53).

### 2.4 Cell culture and treatment

The human DLBCL cell lines OCI-LY3 and SU-DHL-4 were obtained from the DSMZ collection (Germany) and cultured in RPMI-1640 medium (Procell, PM150110) supplemented with 15 % fetal bovine serum (Excell Bio, FSP500) and 1 % penicillin/streptomycin in a 5 % CO2 atmosphere at 37 °C. Cells in the logarithmic phase were collected and transfected with *B2M*-specific siRNA or overexpressing plasmid using Lipofectamine 2,000 (Invitrogen, 52,887) according to the manufacturer’s instructions. The transfection efficiency was then assessed using real-time quantitative polymerase chain reaction (RT-qPCR) and western blot analysis after 24 h of incubation.

### 2.5 RT-qPCR

To validate *B2M* knockdown efficiency, RT-qPCR was conducted on OCI-LY3 cells. Total RNA was isolated using TRIzol™ Reagent (Ambion, #15596-026) followed by DNase I treatment to eliminate genomic DNA contamination. First-strand cDNA synthesis was performed with 1 μg RNA using HiScript^®^ II Q RT SuperMix (Vazyme, R223-01) under the following conditions: 42 °C for 15 min, 85 °C for 5 s. Quantitative PCR amplification was carried out in triplicate using ChamQ Universal SYBR qPCR Master Mix (Vazyme, Q711-02) on a QuantStudio 6 Flex system (Applied Biosystems), with the following cycling parameters: 95 °C for 30 s, 40 cycles of 95 °C for 10 s, and 60 °C for 30 s *B2M* expression levels were normalized to GAPDH and calculated using the comparative 2^−ΔΔCt^ method.

### 2.6 Western blot

Total protein was isolated from OCI-LY3 cells 24 h post-treatment using RIPA lysis buffer (Servicebio, G2002), with protein concentrations determined by BCA assay (GBCBIO, G3522). Equal protein aliquots (20 μg per sample) underwent separation via 10 % sodium dodecyl sulfate-polyacrylamide gel electrophoresis followed by semi-dry electrophoretic transfer to polyvinylidene difluoride membranes (Millipore, IPVH00010). After blocking with 5 % skim milk 2 h at room temperature, the membranes were incubated with primary antibodies against PD-L1 (1:2,000; Proteintech Group, 66248-1-Ig), *B2M* (1:5,000; Proteintech Group, 83683-6-RR), HLA-A (1:5,000; Proteintech Group, 15240-1-AP), HLA-B (1:2,000; Proteintech Group, 17260-1-AP), HLA-C (1:2,000; Proteintech Group, 15777-1-AP), TAP1 (1:1,000; Affinity, DF13735), TAP2 (1: 1,000; Affinity, DF6498), and GAPDH (1:50,000; Proteintech Group, 60004-1-Ig) overnight at 4 °C. Following three 10-min TBST washes, membranes were incubated with HRP-conjugated secondary antibody for 2 h at room temperature. Thereafter, protein signals were detected by enhanced chemiluminescence and quantitatively analyzed using a microplate reader (Bio-Tek), with GAPDH normalization for data standardization.

### 2.7 Flow cytometry

The harvested cells were prepared as a single-cell suspension at a concentration of 1 × 10^7^/mL, and incubated with anti-CD25, anti-CD69, anti-HLA-A, anti-HLA-B, anti-HLA-C, and anti-PD-L1 for 30 min in the darker. After washing, the cells were subjected to a flow cytometry (Beckmancoulter, cytoFLEX).

### 2.8 LDH assay

The LDH release assay was performed using a commercial LDH-cytotoxicity assay kit (Cayman Chemical) according to the manufacturer’s protocol. Briefly, following a 48-h incubation period, 100 μL of supernatant was transferred into a new 96-well plate and mixed with 100 μL of the kit-supplied reaction mixture. After a 30-min incubation at room temperature, absorbance measurements were recorded at 490 nm using a Bio-Tek microplate reader.

### 2.9 Statistical analysis

All *in vitro* experimental data are expressed as the mean ± standard deviation (SD) from three independent replicates (*n* = 3). Statistical analyses were performed using GraphPad Prism 9.0 software. For comparisons between two groups, a two-tailed Student’s *t*-test was applied. For comparisons involving more than two groups, one-way analysis of variance (ANOVA) was conducted, followed by Tukey’s post hoc test for multiple comparisons. Statistical significance was defined as *P* < 0.05.

## 3 Results

### 3.1 Downregulation of *B2M* contributes to PD-L1 expression suppression in DLBCL cells

**Figure 1:**
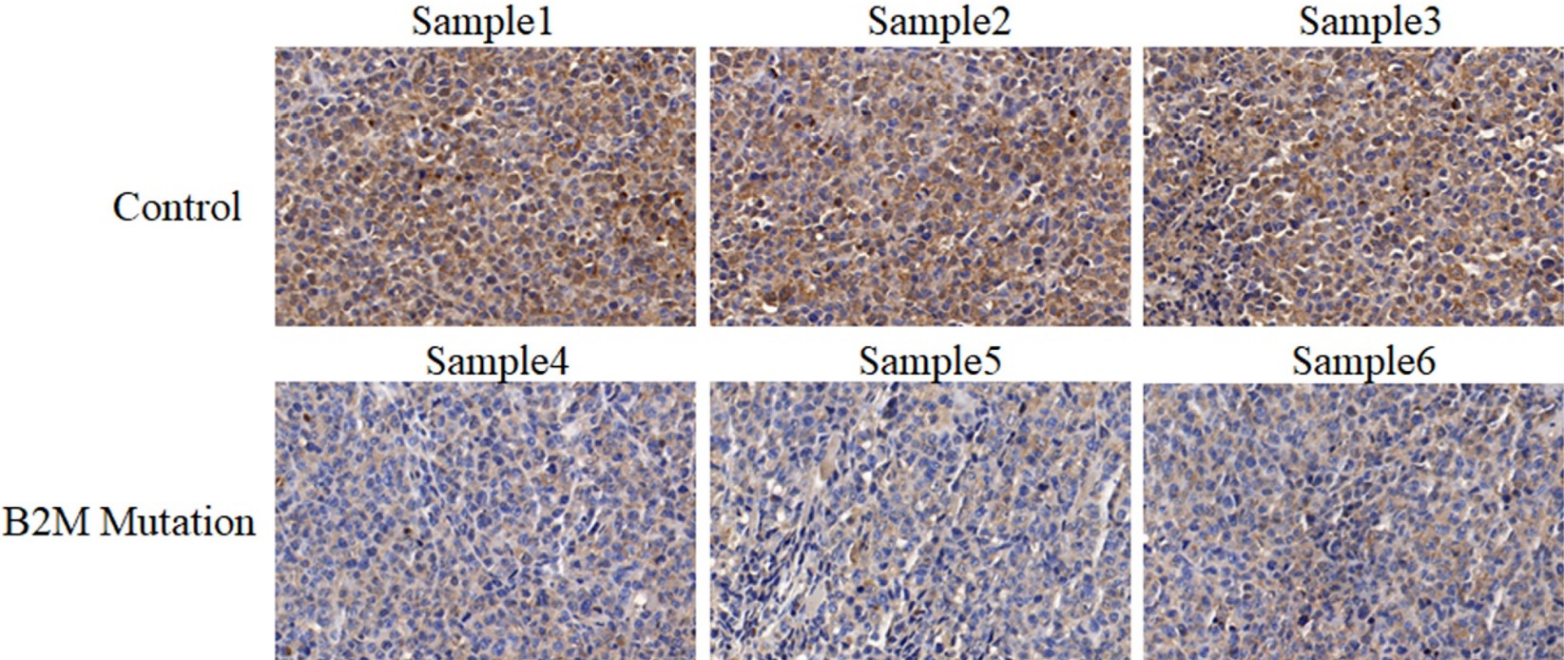
PD-L1 is downregulation in DLBCL patients with *B2M* mutation. The expression of PD-L1 in tissues of DLBCL patients with or without *B2M* mutation was detected using immunohistochemical staining, demonstrating the lower expression of PD-L1 in *B2M* mutation patients. Brown represents the expression of PD-L1. Magnification: × 400.

**Figure 2:**
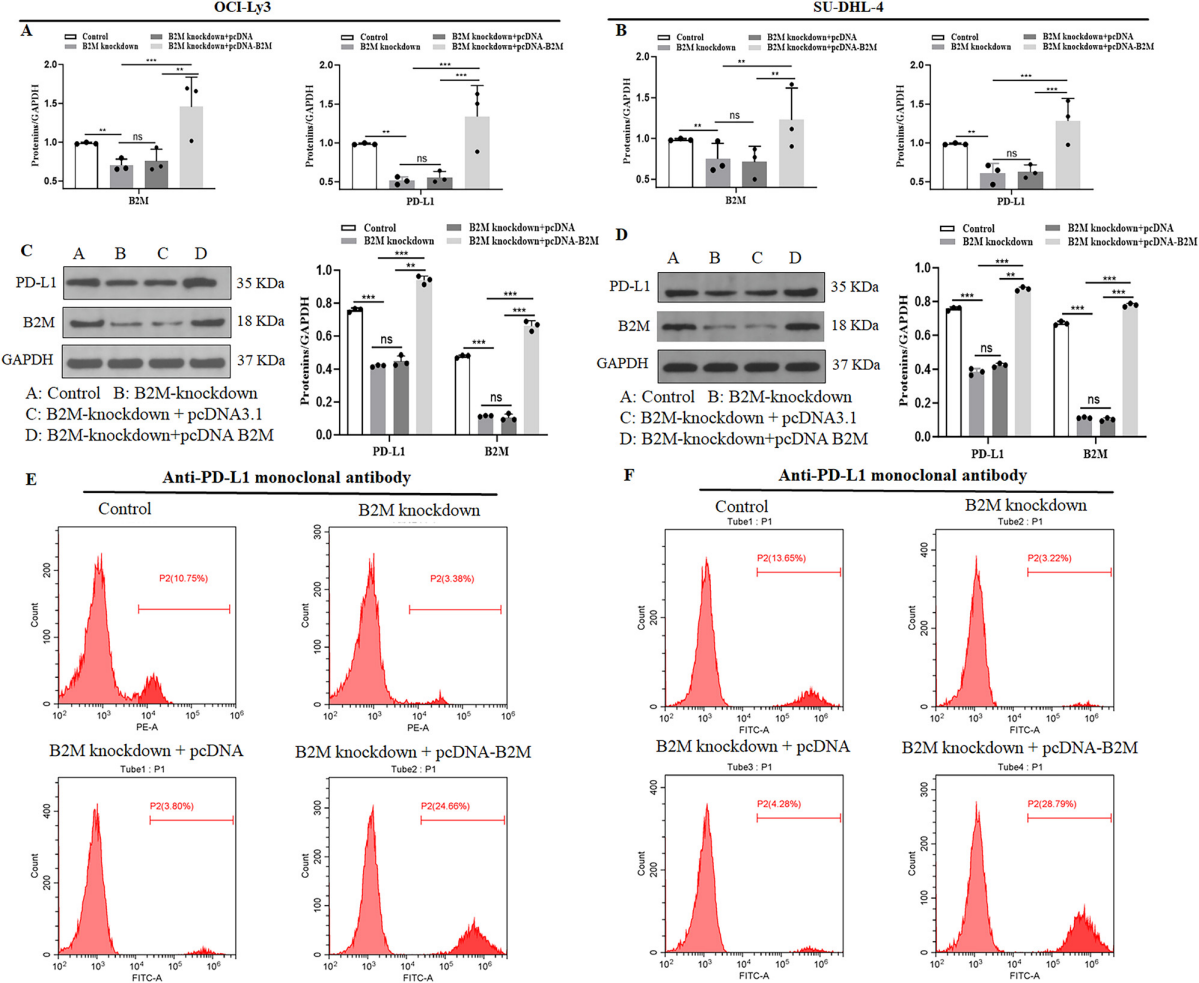
*B2M* knockdown results in downregulation of PD-L1 expression in DLBCL cells. (A–B) real-time quantitative polymerase chain reaction and (C–D) western blot were performed to detect the *B2M* and PD-L1 expressions in OCI-LY3 and SU-DHL-4 cells following *B2M*-specific siRNA or overexpressing plasmid transfection. (E–F) flow cytometry was performed to detect the PD-L1 expression on the surface of OCI-LY3 and SU-DHL-4 cells following *B2M*-specific siRNA or overexpressing plasmid transfection. **p < 0.01, ***p < 0.001. ns, not significant.

As shown in Figure 1, DLBCL patients with *B2M* mutation demonstrated the lower expression of PD-L1 in comparison with patients with *B2M* normal (Control) (Only three representative samples per group are shown out of the total six analyzed.). Further *in vitro* experiments suggested that *B2M* knockdown inhibited PD-L1 mRNA (Figure 2A) and protein (Figure 2B) expressions in both OCI-Ly3 and SU-DHL-4 cell lines. In addition, flow cytometry showed that *B2M* inhibition suppresses PD-L1 expression in the surface of OCI-Ly3 and SU-DHL-4 cells (Figure 2C). The decrease of PD-L1 expression induced by *B2M* inhibition was reversed by *B2M* restoration in DLBCL cells (Figure 2).

### 3.2 *B2M*-knockdown DLBCL cells may escape immune recognition as a result of the loss of MHC-I molecule expression

**Figure 3:**
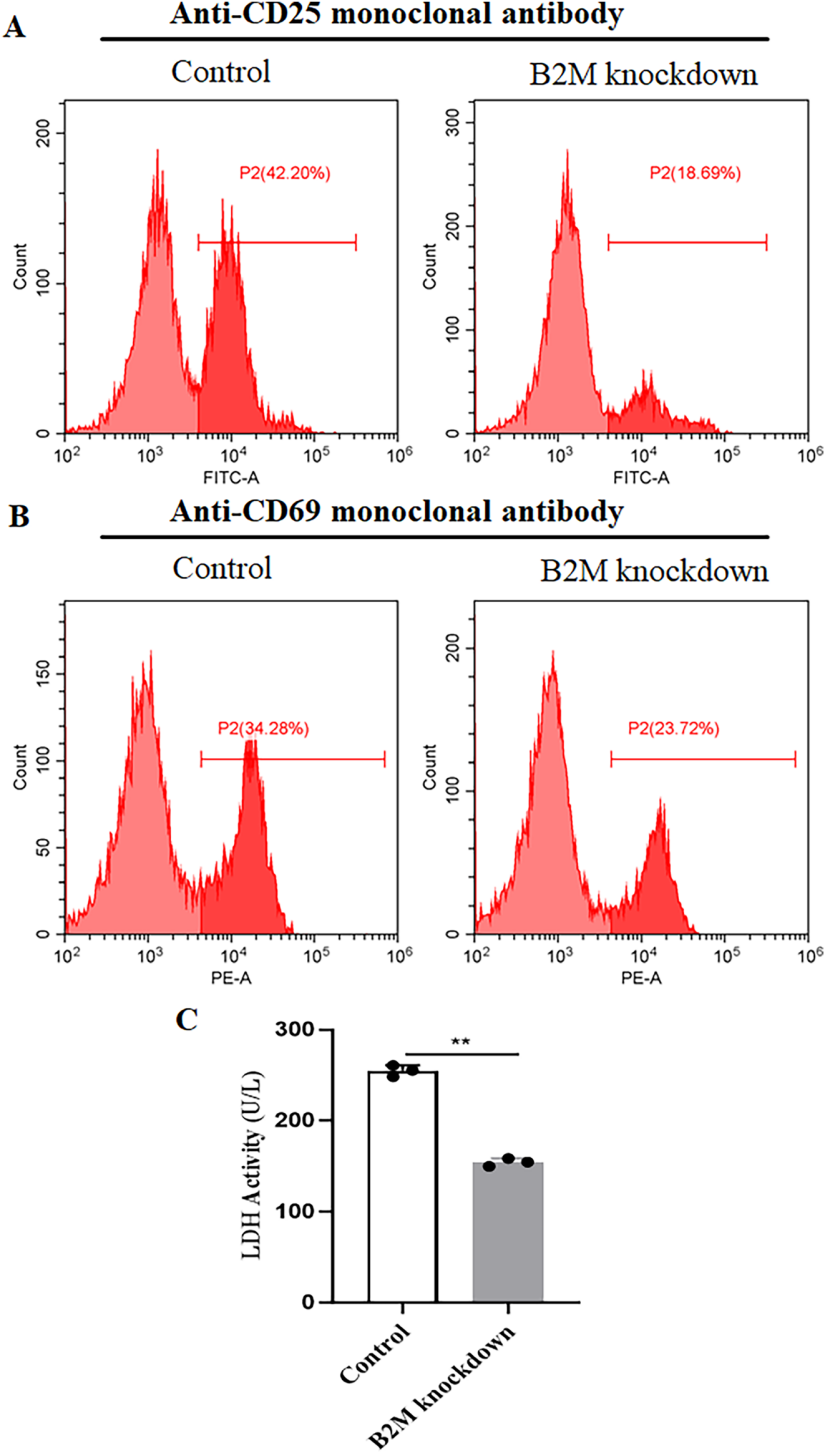
*B2M* knockdown inhibits *T* cell activation. OCI-LY3 cells were co-cultured with human PBMC, (A–B) flow cytometry was then performed to detect the expression of *T* cell activation markers (CD25 and CD69) and (C) lactate dehydrogenase activity was detected in the supernatant. **p < 0.01.

**Figure 4:**
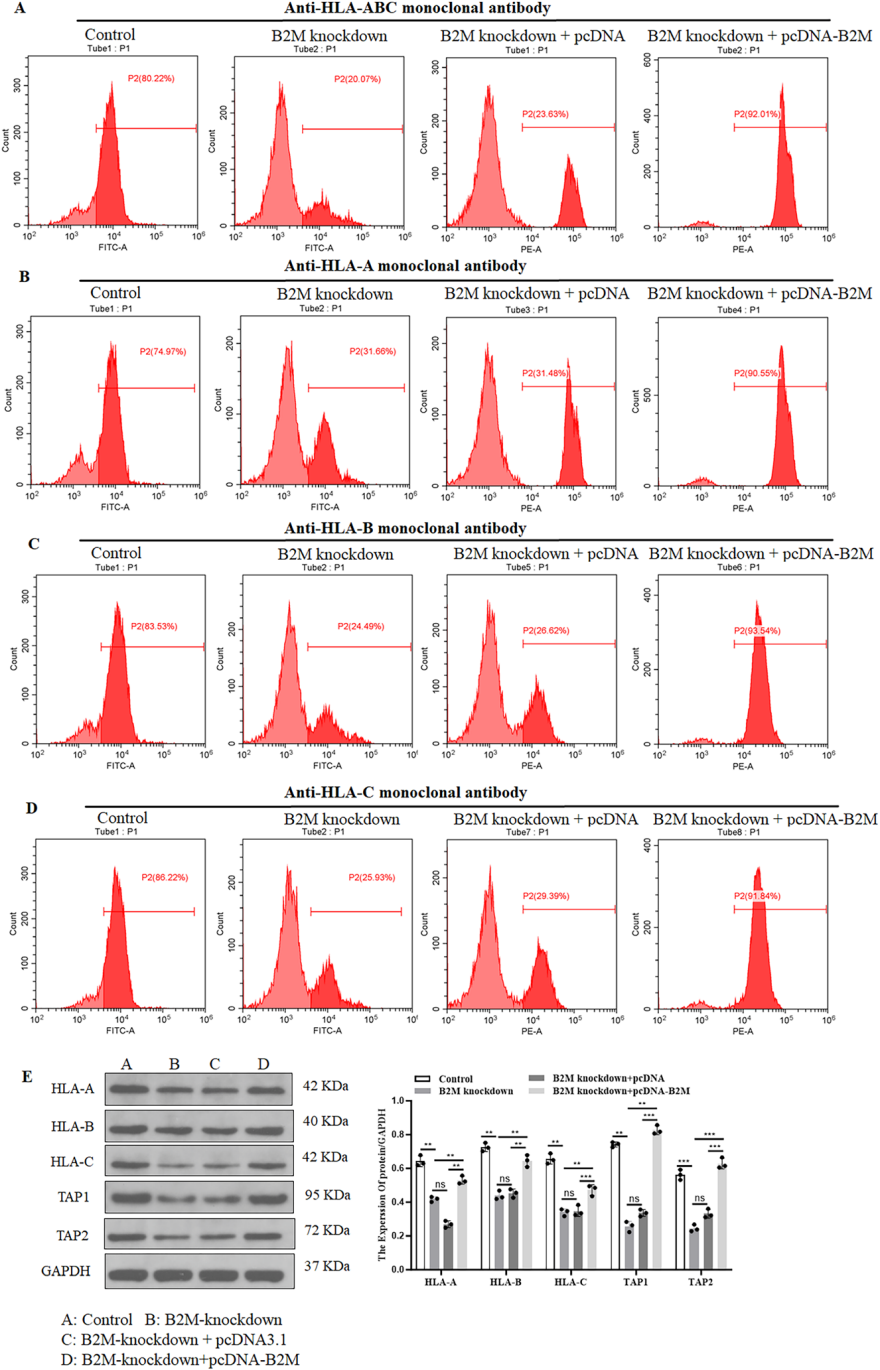
*B2M* knockdown contributes to the decreased expression of the MHC-I complex in DLBCL cells. Flow cytometry was performed to detect the expression level of (A) HLA-ABC, (B) HLA-A, (C) HLA-B, and (D) HLA-C in OCI-LY3 cells following *B2M*-specific siRNA or overexpressing plasmid transfection. (E) Westen blot was performed to detect the proteins expression of HLA-A, HLA-B, HLA-C, TAP 1, and TAP2 in OCI-LY3 cells following *B2M*-specific siRNA or overexpressing plasmid transfection. **p < 0.01, ***p < 0.001. ns, not significant.

To investigate the impact of *B2M* on immune response, we co-cultured OCI-Ly3 cells with human PBMC. The analysis revealed that *B2M* knockdown in DLBCL cells led to reduced expression of *T* cell activation markers (CD25, Figure 3A; CD69, Figure 3B) and decreased LDH release in the supernatant (Figure 3C) compared to controls. Moreover, we observed the downregulation of HLA-ABC (Figure 4A), HLA-A (Figure 4B), HLA-B (Figure 4C), and HLA-C (Figure 4D), in *B2M* knockdown DLBCL cells. Western blot assay further validated that *B2M* knockdown resulted in the suppression of HLA-A, HLA-B, HLA-C, TAP1, and TAP2 proteins levels in DLBCL cells (Figure 4E). These results suggesting that *B2M*-deficient DLBCL cells may evade immune surveillance through impaired MHC-I molecule expression, resulting in suppressed T-cell activation.

### 3.3 *B2M* knockdown drives PD-1 inhibitor resistance in DLBCL through dual immune escape mechanisms

**Figure 5:**
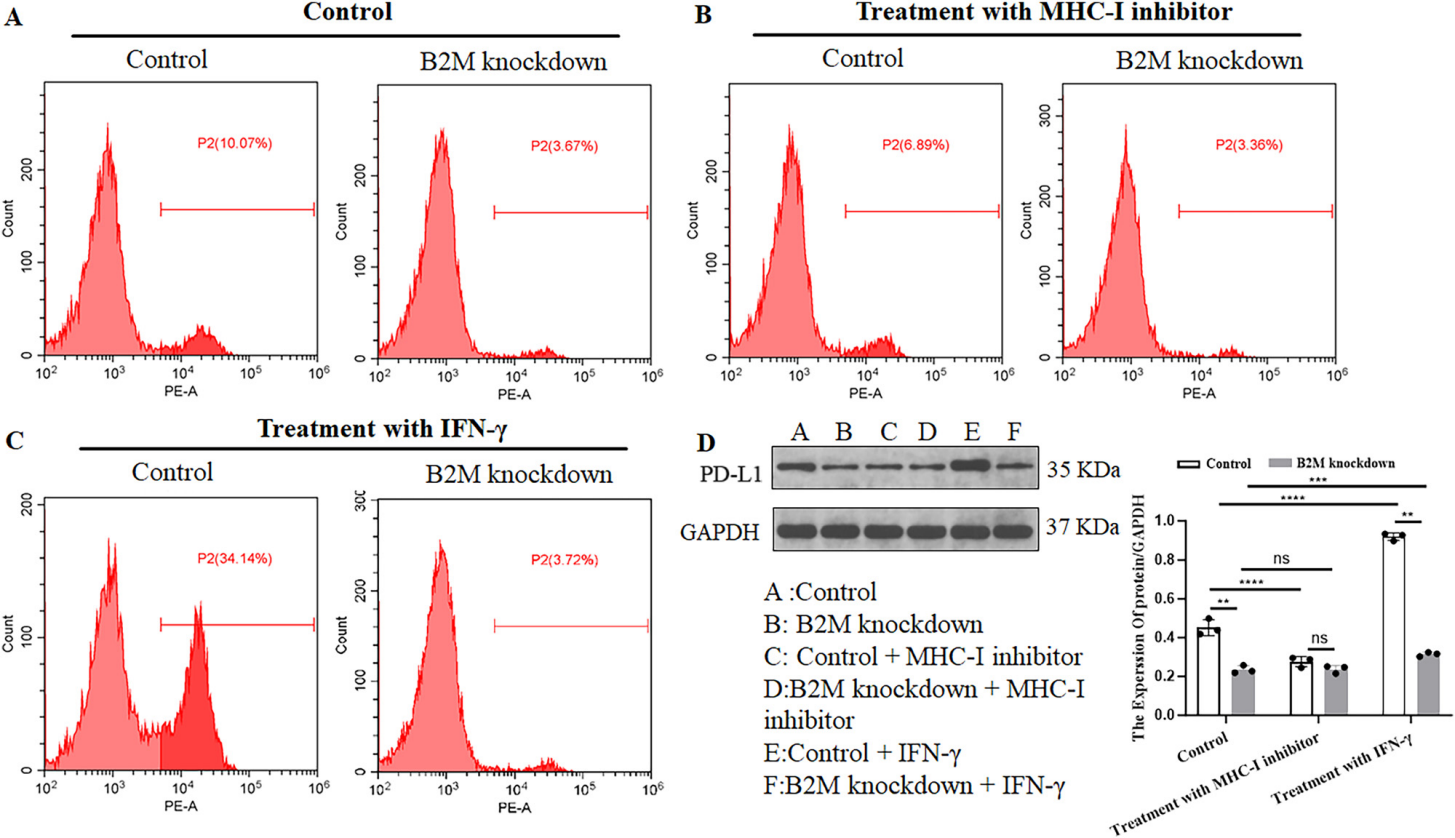
*B2M* knockdown drives PD-1 inhibitor resistance in DLBCL through dual immune escape mechanisms. (A) Flow cytometry was performed to detect the PD-L1 expression level in OCI-LY3 cells following *B2M*-specific siRNA transfection in conbinaition with/without MHC-I inhibition or IFN-γ treatment. (B) Western blot was performed to detect the PD-L1 expression in OCI-LY3 cells following *B2M*-specific siRNA transfection in conbinaition with/without MHC-I inhibition or IFN-γ treatment. **p < 0.01, ***p < 0.001, ****p < 0.0001. ns, not significant.

**Figure 6:**
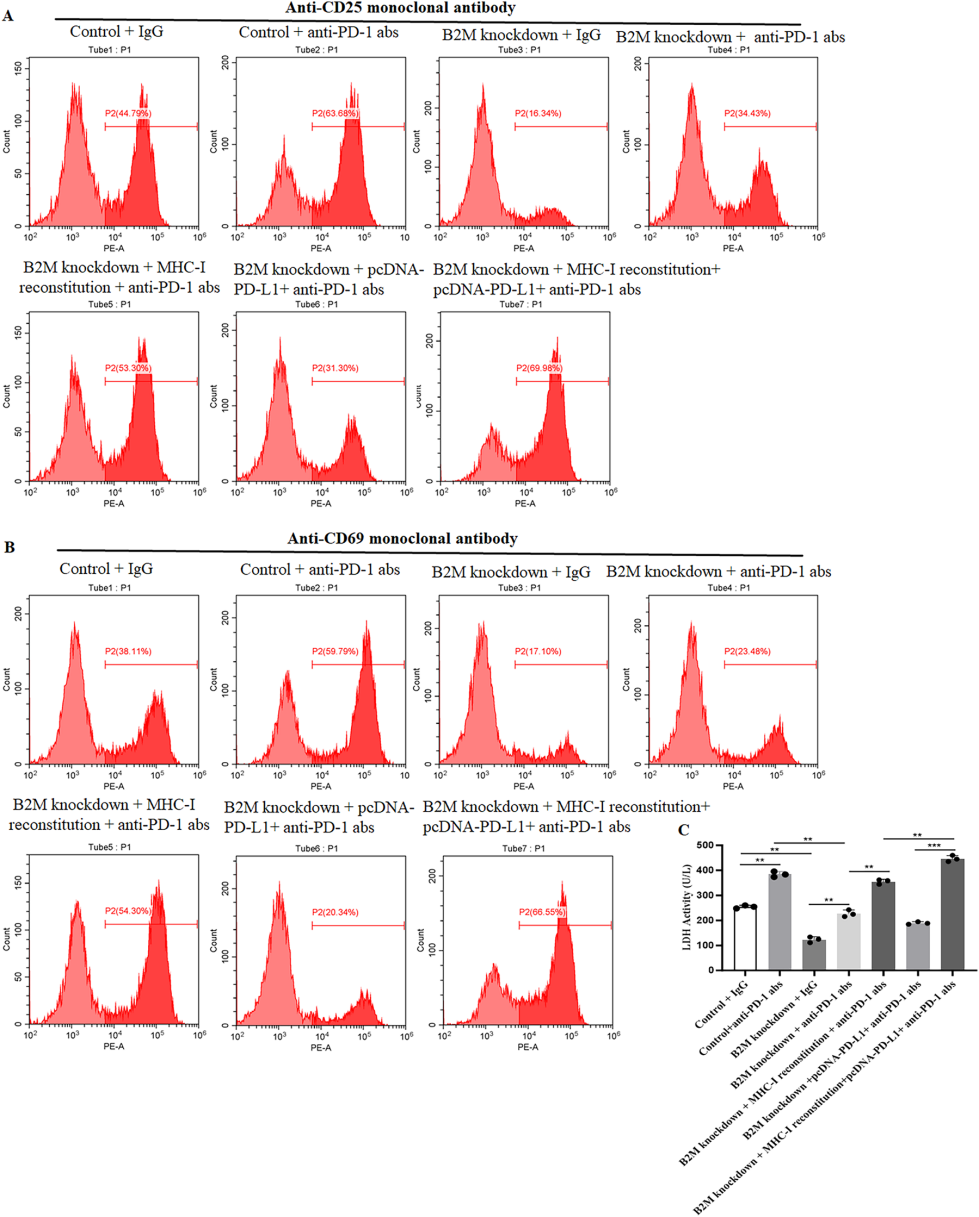
Functional validation of *B2M*-mediated PD-1 inhibitor resistance and reversal strategies. OCI-LY3 cells with different treatment were co-cultured with human PBMC, (A–B) flow cytometry was then performed to detect the expression of *T* cell activation markers (CD25 and CD69) and (C) lactate dehydrogenase activity was detected in the supernatant. **p < 0.01, ***p < 0.001.

To investigate whether the *B2M* knockdown-induced PD-L1 inhibition is associated with MHC-I suppression, we treated OCI-Ly3 cells with MHC-I inhibitor and IFN-γ, whose stimulates is usually found to enhance the expression of MHC-I [11]. Flow cytometry demonstrated that, in *B2M* mutated DLBCL cells, the expression of PD-L1 was inhibited in comparison with normal DLBCL cells, and the combination treatment with MHC-I inhibitor or IFN-γ can not influence the PD-L1 expression (Figure 5A). However, MHC-I inhibitor decreased PD-L1 level, while IFN-γ increased PD-L1 level in normal DLBCL cells (Figure 5A). The results of flow cytometry were further confirmed by western blot assay (Figure 5B). These results indicated that, in DLBCL cells, the expression of MHC-I is correlated with PD-L1 expression. Disruption of antigen presentation function induced by *B2M* knockdown is associated with reduced PD-L1 expression. In other words, intact MHC-I function is a prerequisite for PD-L1 expression. In addition, we co-cultured wild-type and *B2M*-knockdown DLBCL cells with human PBMCs, adding clinically relevant doses of anti-PD-1 antibodies (10 μg/mL) along with MHC-I reconstitution or PD-L1 overexpression vectors to validate the impact of *B2M* on PD-1 resistance and its underlying mechanisms. As shown in Figure 6A–C, the anti-PD-1 antibody significantly enhanced *T* cell activation (increased proportion of CD69+/CD25+ *T* cells) and tumor cell killing (elevated LDH release) in wild-type cells. In contrast, *B2M* knockdown cells failed to elicit effective immune responses, with markedly reduced *T* cell activation and cytotoxicity, directly demonstrating functional resistance. Notably, MHC-I restoration substantially mitigated the impairment in *T* cell activation and cytotoxicity caused by *B2M* knockdown. While elevated PD-L1 expression alone had no significant effect on the diminished immune killing induced by *B2M* knockdown, the combination of MHC-I restoration with PD-L1 overexpression significantly reversed this effect, exhibiting superior efficacy compared to MHC-I restoration alone. These results further underscore the synergistic role of MHC-I and PD-L1 in modulating PD-1 resistance mediated by *B2M*.

## 4 Discussion

The mechanisms of tumor immune escape can be primarily categorized into: weak immunogenicity of tumor cells, antigen modulation or antigen loss in tumor cells, absence of co-stimulatory signals, tumor-induced immunosuppressive effects, and the formation of immune-privileged sites induced by tumors [12], 13]. Based on these escape mechanisms, various therapeutic approaches with distinct mechanisms of action have been developed. ICIs (e.g. anti-CTLA-4 monoclonal antibodies, anti-PD-1 monoclonal antibodies, and anti-PD-L1 monoclonal antibodies) exert antitumor activity by alleviating immune suppression within the tumor microenvironment and reactivating immune cells [7]. Immune checkpoint molecules refer to a class of molecules expressed on immune cells that function to suppress immune responses. During tumor progression, these molecules emerge as one of the primary contributors to immune tolerance. By upregulating immune checkpoints, tumor cells can effectively inhibit immune reactions, thereby enabling their evasion from immune-mediated elimination. PD-1 stands as one of the relatively well-characterized immune checkpoint molecules in current research [14]. PD-L1 serves as the ligand for PD-1. The binding of PD-L1 expressed on tumor cells to PD-1 receptors on *T* cells attenuates TCR signal transduction. Prolonged antigen stimulation leads to *T* cell dysfunction and exhaustion, ultimately facilitating tumor immune evasion. Blockade PD-1 on *T* cell surfaces inhibits PD-L1/PD-1 interactions, thereby promoting *T* cell activation, triggering antitumor immune responses, remodeling the tumor microenvironment, and consequently suppressing immune evasion mechanisms to restrict tumor growth [15]. Clinical studies demonstrate that PD-1 inhibitors exhibit significant therapeutic efficacy in DLBCL patients, particularly within PD-L1 high-expressing subtypes such as EBV-positive [16] and primary mediastinal variants [17], 18].

While ICIs have revolutionized cancer treatment by significantly enhancing clinical outcomes across diverse malignancies, their therapeutic potential remains constrained by two critical barriers: primary resistance (failure to achieve initial response) and acquired resistance (disease progression after transient benefit). Even in melanoma-a malignancy with the highest reported objective response rates to ICIs – 60–70 % of patients exhibit primary resistance to anti-PD-1 monotherapy; Among initial responders, 20–30 % inevitably develop tumor relapse through acquired resistance mechanisms, underscoring the evolutionary adaptability of malignancies under immune pressure [7]. A multicenter study involving 109 patients with peripheral *T*-cell lymphoma demonstrated that JAK3 mutation-induced downregulation of PD-L1 expression is associated with poor prognosis in patients receiving PD-1 antibody therapy [19]. Yang et al. further demonstrated that genome-wide CRISPR-mediated CD28 knockdown reversed anti-PD-1 resistance in triple-negative breast cancer (TNBC) murine models, while clinical analyses identified CD28 upregulation in TNBC tissues as being correlated with elevated PD-L1 expression and poorer prognosis [20]. These suggests the effect of PD-L1 expression in acquired resistance of PD-1 inhibitor. In this work, we revealed that *B2M* mutations significantly downregulate PD-L1 expression, suggesting that *B2M* deficiency may drive PD-1 inhibitor resistance in DLBCL patients through impaired PD-L1 expression.

Moreover, we observed that *B2M* mutation induced MHC-I expression suppression, as demonstrated by the decrease of HLA-A, HLA-B, HLA-C, TAP1, and TAP2 [21]. In addition, we found that *B2M*-deficient cells failed to activate CD8^+^ *T* cells, as evidenced by diminished CD69^+^/CD25^+^ surface expression and reduced cytotoxic efficiency. The MHC-I processing machinery plays a pivotal role in orchestrating CD8^+^ *T* cell -mediated antitumor responses, where its functional impairment is mechanistically associated with diminished tumor immunogenicity and immunotherapy resistance [22]. Evidence have revealed that post-relapse tumor specimens exhibited a *B2M* frameshift mutation that abolished MHC-I surface expression, enabling immune escape from CD8^+^ *T* cell surveillance [9]. This parallels lung cancer study where MHC I presentation defects correlate with acquired immunotherapy resistance, suggesting targetable vulnerabilities in antigen processing pathways [23].

Furthermore, we found that disruption of antigen presentation function induced by *B2M* knockdown is associated with reduced PD-L1 expression, namely, intact MHC-I function is a prerequisite for PD-L1 expression. Through incorporating co-culture systems with PD-1 blockade, we further validated the functional roles of MHC-I and PD-L1 in modulating PD-1 inhibitor resistance mediated by *B2M*. Current research on the regulatory interplay between MHC-I and PD-L1 in immune responses remains limited. Tumor-specific CD8^+^ *T* cell activation is critically dependent on the efficient recognition of MHC-I-presented tumor antigens [22], with subsequent IFN-γ secretion by activated CD8^+^ *T* cells driving PD-L1 upregulation on tumor cells [11], 24]. This suggests that the efficacy of anti-PD-L1/PD-1 therapies may be intrinsically linked to the initial CD8^+^ *T* cell-tumor cell interaction. Thus, we speculate that *B2M* mutation-induced resistance to PD-1 inhibitors could arise through a mechanistic cascade involving impaired antigen presentation, defective CD8^+^ *T* cell priming, and consequent downregulation of PD-L1 expression – a proposed mechanism requiring systematic validation through integrated *in vitro* and *in vivo* experimental approaches.

In conclusion, the current study mechanistically linked *B2M* mutations to co-suppression of PD-L1 and MHC-I in DLBCL, revealing a dual-pathway immune evasion strategy – inhibition of PD-L1 expression and disruption of antigen presentation function. This bifurcated mechanism may synergistically drive resistance to PD-1 axis inhibitors. However, this study has several limitations. We only examined the effects of *B2M* on PD-L1 and MHC-I expression and the preliminary drug sensitivity but did not conduct further animal validation. In addition, the limited sample size may compromise the statistical robustness of our findings. Future research will involve prospective studies incorporating animal experiments and expanded clinical cohort to validate and complement these findings. Collectively, this work provides a theoretical foundation for developing combination strategies to enhance immunotherapy efficacy in DLBCL.
